# Environment-dependent responses of soil physicochemical properties, microbial communities and functional gene profiles to protected and open-field cultivation of *Stropharia rugosoannulata*

**DOI:** 10.3389/fmicb.2026.1912245

**Published:** 2026-10-14

**Authors:** Wei Ji, Xueyan Tang, Xiaomei Liu, Wenying Su, Wenbo Chai, Kelong Chen, Jintao Ge, Baoxiang Wang

**Affiliations:** 1 Lianyungang Academy of Agricultural Sciences, Lianyungang, China; 2 Qinghai Province Key Laboratory of Physical Geography and Environmental Process, College of Geographical Science, Qinghai Normal University, Xining, China

**Keywords:** antibiotic resistance genes, metagenomics, protected cultivation, soil microbiome, *Stropharia rugosoannulata*

## Abstract

The cultivation of *Stropharia rugosoannulata* involves substantial organic substrate input and fungal mycelial activity, which may influence soil nutrient transformation, microbial communities and functional gene profiles. However, whether these responses differ between protected and open-field cultivation settings remains unclear at the metagenomic level. In this study, four treatments were investigated: protected cultivation soil (NT), protected uncultivated soil (NK), open-field cultivation soil (ZT) and open-field uncultivated soil (ZK). Soil physicochemical properties, enzyme activities and shotgun metagenomes were analyzed to compare cultivation-associated soil responses under the two sampled cultivation settings. Protected cultivation showed more evident biochemical responses than open-field cultivation. Compared with NK, NT had significantly higher ${\text{NH}}_{4}^{\text{+}}$-N content and sucrase activity, together with lower soil salinity and electrical conductivity, whereas no significant differences in the measured soil properties were detected between ZT and ZK. Exploratory two-way ANOVA indicated setting-associated variation in several soil variables and significant interaction effects for ${\text{NH}}_{4}^{\text{+}}$-N and ${\text{NO}}_{3}^{-}$-N. Metagenomic analysis showed treatment-associated variation in specific classified genera, with *Cellvibrio* and *Flavobacterium* showing higher relative abundances in cultivated soils. However, PERMANOVA did not detect significant separation in the overall genus-level community structure. KEGG functional profiles remained broadly stable across treatments, although protected cultivation showed more evident variation in selected functional categories. Antibiotic resistance gene profiles also exhibited treatment-specific patterns. Overall, *S. rugosoannulata* cultivation did not induce uniform microbial community restructuring or consistent soil improvement, but was associated with context-dependent responses in soil biochemical properties, specific microbial taxa, functional potential and resistome composition. These findings provide metagenomic insights for soil health management and resistome monitoring in continuous edible mushroom cultivation systems.

## 1 Introduction

*Stropharia rugosoannulata* Farl. ex Murrill is an important edible mushroom with strong lignocellulose-degrading capacity. It can utilize agricultural organic wastes, such as straw, sawdust and spent mushroom substrate, as cultivation substrates, and therefore has considerable value for edible mushroom production, agricultural waste recycling and soil improvement (Huang et al., 2023; Luo et al., 2023). In recent years, with the expansion of *S. rugosoannulata* cultivation, several cultivation systems have been developed, including paddy-field ecological cultivation, understory ecological cultivation and protected cultivation in greenhouse facilities (Bian, 2023; Wu et al., 2024). These cultivation environments differ markedly. Protected cultivation generally provides relatively stable temperature and moisture conditions and greater management controllability, whereas open-field cultivation is more directly affected by rainfall, light, temperature fluctuations and background soil conditions (Yang X. B. et al., 2020; Guo et al., 2025). Such environmental differences may regulate substrate decomposition, nutrient release and the soil microenvironment, thereby further influencing the structure and function of soil microbial ecosystems. Previous studies have shown that *S. rugosoannulata* cultivation can alter soil nutrient status and bacterial community composition, and that residual cultivation substrates can have important effects on soil nutrient supply and microbial communities (Hao et al., 2024; Liu et al., 2025).

Soil microorganisms are key components maintaining the functioning of agricultural ecosystems and are widely involved in organic matter decomposition, carbon, nitrogen and phosphorus cycling, nutrient transformation, plant growth regulation and soil health maintenance (Gao et al., 2024; Li et al., 2025). The structure and function of soil microbial communities are jointly shaped by soil physicochemical properties, resource availability, vegetation or cultivation practices and external environmental conditions (Waymouth et al., 2020; Edwards et al., 2023). During edible mushroom cultivation, fungal mycelial growth, substrate degradation, organic carbon input and nutrient release may all alter soil microbial community composition and functional potential. In particular, *S. rugosoannulata* cultivation is usually accompanied by the incorporation of large amounts of organic substrates rich in cellulose, hemicellulose and lignin into the soil system. This process may affect soil organic matter, inorganic nitrogen, available phosphorus, available potassium and salinity, and may also change soil enzyme activities associated with carbon transformation and redox processes (Gong et al., 2018; Tang et al., 2022; Guo et al., 2023).

Current studies on the soil ecological effects of edible mushroom cultivation have mainly focused on the recycling of cultivation substrates, improvement of soil nutrients, yield enhancement, or bacterial community changes based on amplicon sequencing. Mushroom spent substrate or cultivation residues have been reported to act as soil amendments, influencing soil organic carbon, nitrogen mineralization, microbial communities and functional diversity (Lou et al., 2017; Gong et al., 2018; Li et al., 2020; Hu et al., 2021). Field-based edible mushroom cultivation may also promote the cycling of soil carbon, nitrogen, phosphorus and potassium through mycelial activity and substrate decomposition (Hu et al., 2021; Tang et al., 2022). However, several knowledge gaps remain. First, comparative studies on the effects of *S. rugosoannulata* on soil microbial ecosystems under protected and open-field cultivation environments are still limited. Second, most studies have focused primarily on soil physicochemical properties or microbial taxonomic composition, whereas metagenomic-level functional gene composition and metabolic potential remain insufficiently resolved. Third, whether *S. rugosoannulata* cultivation affects the soil antibiotic resistance gene pool and its potential ecological indicator value remains poorly understood.

Metagenomic sequencing can provide information beyond microbial taxonomic composition by resolving soil microbial functional gene profiles, metabolic pathway potential and antibiotic resistance gene spectra, thereby offering a more comprehensive understanding of how agricultural management practices influence soil microbial ecosystems. Previous studies have demonstrated that metagenomic approaches can simultaneously reveal soil microbial taxonomic composition, functional attributes, nutrient cycling-related genes and antibiotic resistance genes (Fierer et al., 2012). In agricultural soils, antibiotic resistance genes (ARGs) are important components of the environmental microbiome resistome. Soil microorganisms are considered important reservoirs of diverse resistance genes, and ARG composition may be jointly affected by fertilization regime, organic fertilizer or manure input, soil physicochemical properties, microbial community structure and environmental factors (Riesenfeld et al., 2004; Wright, 2007; Nesme et al., 2014). Therefore, when evaluating the soil ecological effects of agricultural management practices such as edible mushroom cultivation, simultaneous analysis of ARG composition and treatment-specific variation, in addition to nutrient cycling and microbial functions, can help provide a more comprehensive assessment of soil microbial ecological safety and potential environmental risks (Wang et al., 2020; Liu et al., 2024).

Based on these considerations, this study investigated four treatments: protected *S. rugosoannulata* cultivation soil (NT), protected uncultivated soil (NK), open-field *S. rugosoannulata* cultivation soil (ZT) and open-field uncultivated soil (ZK). Soil physicochemical properties, enzyme activities and shotgun metagenomic data were integrated to compare cultivation-environment-dependent soil responses under protected and open-field conditions. The specific objectives were to: (1) compare the effects of *S. rugosoannulata* cultivation on soil nutrients, salinity and enzyme activities under protected and open-field conditions; (2) characterize soil microbial community composition, differential marker taxa and KEGG functional gene profiles across treatments; (3) describe ARG composition and differential enrichment patterns under different cultivation treatments; and (4) conduct exploratory ordination to evaluate potential associations between soil physicochemical properties, enzyme activities and dominant bacterial genera. The results provide evidence that soil biochemical properties, microbial taxonomic composition, functional potential and ARG profiles may respond differently to *S. rugosoannulata* cultivation depending on the cultivation environment, and may provide useful information for soil health management in edible mushroom cultivation systems.

## 2 Materials and methods

### 2.1 Experimental design and soil sampling

Two cultivation systems of *Stropharia rugosoannulata*, namely protected cultivation and open-field cultivation, were investigated in this study, with corresponding uncultivated soils used as controls. The *S. rugosoannulata* cultivar AF-1, independently developed by the Lianyungang Academy of Agricultural Sciences and maintained in its fungal germplasm collection, was used for cultivation. The experiment was conducted at the Dongxin Experimental Base of the Lianyungang Academy of Agricultural Sciences, Lianyungang, Jiangsu Province, China. Cultivation was carried out from December to April of the following year, and soil samples were collected in May after completion of the cultivation cycle. Four treatments were established: protected *S. rugosoannulata* cultivation soil (NT), protected uncultivated soil (NK), open-field *S. rugosoannulata* cultivation soil (ZT), and open-field uncultivated soil (ZK). Each treatment included three biological replicates, resulting in a total of 12 soil samples. The protected and open-field plots represented two sampled cultivation settings, each with its corresponding uncultivated control soil; therefore, within-setting comparisons, namely NT vs. NK and ZT vs. ZK, were used as the primary comparisons for evaluating cultivation-associated soil responses. *S. rugosoannulata* was cultivated using rice straw as the cultivation substrate. The straw was laid to a thickness of approximately 30 cm, the spawn was inoculated by broadcasting, and the cultivation beds were subsequently covered with approximately 2 cm of soil. No fertilizer, pesticide, fungicide, or antibiotic was applied during cultivation. During the fruiting period, water was sprayed twice per week for approximately 5 min each time to maintain moisture in the cultivation beds. Soil samples were collected from beneath the cultivation substrate after completion of *S. rugosoannulata* cultivation.

Soil samples were collected from the 0–20 cm soil layer. For each replicate, five subsamples were collected using a five-point sampling method and thoroughly mixed to form one composite sample. Visible plant residues, stones, large mycelial fragments and substrate residues were removed after sampling. Each composite sample was divided into three portions. One portion was air-dried and passed through a 2-mm sieve for the determination of soil physicochemical properties; one portion was stored at 4 °C for soil enzyme activity assays; and the remaining portion was immediately frozen in liquid nitrogen and stored at −80 °C for metagenomic DNA extraction.

### 2.2 Determination of soil physicochemical properties and enzyme activities

Soil physicochemical properties were determined according to established Chinese agricultural and environmental analytical standards. Soil organic matter (SOM) was determined by the potassium dichromate oxidation method according to NY/T 1121.6-2006. Ammonium nitrogen (${\text{NH}}_{4}^{+}$-N) and nitrate nitrogen (${\text{NO}}_{3}^{-}$-N) were extracted with KCl solution and determined colorimetrically according to HJ 634-2012. Available phosphorus (A-P) was determined by spectrophotometry after chemical extraction according to NY/T 1121.7-2014. Available potassium (A-K) was extracted with neutral 1 mol L^−1^ ammonium acetate and determined using flame photometry according to NY/T 889-2004. Soil pH was measured potentiometrically in a soil–water suspension at a soil-to-water ratio of 1:2.5 (w/v) according to NY/T 1121.2-2006. Soil electrical conductivity (EC) was measured using a conductivity meter in a 1:5 (w/v) soil–water extract according to HJ 802-2016. Soil salinity (SAL) was estimated from the conductivity of the soil extract based on the conductivity–salinity conversion used in the analytical procedure.

Soil enzyme activities measured in this study included sucrase (SUC) and catalase (CAT). SUC was selected as an indicator of carbohydrate decomposition and soil organic carbon transformation, while CAT was used to reflect soil oxidative stress response and microbial metabolic activity. Both enzyme activities were measured using commercial assay kits supplied by Suzhou Grace Biotechnology Co., Ltd. The kit numbers for catalase and sucrase were G0303F48 and G0302F48, respectively. All procedures were performed according to the manufacturer's instructions. Sucrase activity was expressed as the amount of reducing sugar produced per unit soil mass per unit time, whereas catalase activity was expressed as the amount of substrate decomposed per unit soil mass per unit time. Three biological replicates were included for all measurements (Ji et al., 2024; Chai et al., 2026). It should be noted that enzymes directly involved in nitrogen and phosphorus transformation, such as urease, protease and phosphatase, were not assayed in this study; therefore, the enzyme activity results mainly reflect carbon transformation-related activity and oxidative stress response rather than direct N- or P-cycling enzyme processes.

### 2.3 Soil metagenomic DNA extraction and sequencing

An appropriate amount of soil sample stored at −80 °C was used for total microbial DNA extraction with a commercial soil DNA extraction kit, following the manufacturer's instructions. DNA integrity was assessed by agarose gel electrophoresis, and DNA purity and concentration were determined using a NanoDrop spectrophotometer and/or a Qubit fluorometer. DNA samples that met the quality requirements were used for metagenomic sequencing library construction. After library preparation, paired-end sequencing was performed on an Illumina high-throughput sequencing platform. Raw sequencing data were obtained from 12 soil metagenomic samples and used for subsequent quality control, assembly, gene prediction, taxonomic annotation and functional annotation analyses (Li et al., 2015; Ji et al., 2025).

The raw metagenomic sequencing data generated in this study have been deposited in the National Microbiology Data Center (NMDC) under project accession number NMDC10020845 and submission number SUB1783416151777. The sample-specific accession numbers are NMDC20596832 (NT1), NMDC20596833 (NT2), NMDC20596834 (NT3), NMDC20596835 (NK1), NMDC20596836 (NK2), NMDC20596837 (NK3), NMDC20596838 (ZT1), NMDC20596839 (ZT2), NMDC20596840 (ZT3), NMDC20596841 (ZK1), NMDC20596842 (ZK2), and NMDC20596843 (ZK3).

### 2.4 Quality control, assembly and construction of the non-redundant gene catalog

Raw sequencing reads were subjected to quality control to remove adapter sequences, low-quality reads, reads with a high proportion of ambiguous bases and other unqualified sequences, thereby generating high-quality clean reads. Clean reads from each sample were first assembled individually using MEGAHIT. Bowtie2 was then used to map the clean reads back to the contigs assembled from each sample, and the unmapped paired-end reads from all samples were pooled for co-assembly using MEGAHIT. Contigs longer than 500 bp were retained for subsequent analyses. Open reading frames (ORFs) were predicted using MetaGeneMark, and predicted coding sequences longer than 100 nt were retained. Predicted gene sequences from all samples were clustered and dereplicated using CD-HIT with a sequence identity threshold of 95% and coverage threshold of 90% to construct a non-redundant gene catalog. Subsequently, gene abundance in each sample was estimated against the non-redundant gene catalog using Salmon. Gene abundance was normalized using transcripts per million (TPM) for downstream comparative analyses (Nielsen et al., 2014; Li et al., 2015). Sequencing quality was assessed using clean reads, clean bases, Q20, Q30 and data utilization rate after quality filtering. Assembly quality was evaluated using QUAST based on contig number, N50, total assembly length and related assembly metrics. These quality-control and assembly metrics were used to evaluate data reliability.

### 2.5 Microbial taxonomic annotation and community structure analysis

The non-redundant gene catalog was aligned against the NCBI non-redundant protein database (NCBI NR; downloaded on 16 May 2022) using DIAMOND blastp with an e-value threshold of 1e^−5^ to obtain microbial taxonomic annotations. Taxonomic assignments were generated based on the best-supported hits using the lowest common ancestor approach. Based on the annotation results, microbial community composition was summarized at different taxonomic levels, with this study focusing on differences at the genus level. Relative abundance bar plots, clustering heatmaps and bubble plots were used to visualize the composition of dominant microbial taxa among treatments. Principal coordinate analysis (PCoA) based on Bray–Curtis dissimilarity was performed to evaluate differences in microbial community structure among treatments. Permutational multivariate analysis of variance (PERMANOVA) was used to test the significance of community structural differences among groups. Linear discriminant analysis effect size (LEfSe) was applied to identify differential marker taxa among treatments, with *p* < 0.05 and LDA score >3.0 used as the significance thresholds for microbial taxa (Karlsson et al., 2013; Buchfink et al., 2015).

### 2.6 KEGG functional annotation and functional composition analysis

The non-redundant gene catalog was aligned against the Kyoto Encyclopedia of Genes and Genomes database (KEGG; Release 96, downloaded on 15 December 2020) using DIAMOND blastp with an e-value threshold of 1e^−5^ to obtain gene function and metabolic pathway annotations. The best-scoring hit was used to assign KEGG orthologs and pathway information to each gene. Based on normalized gene abundance, the relative abundances of KEGG functional categories and pathways were calculated for each sample. Differences in microbial functional composition among treatments were compared at the KEGG Level 2 and pathway levels. Stacked bar plots were used to show KEGG functional composition across samples, and box plots were used to compare significantly different KEGG Level 2 functional categories between NT and NK, and between ZT and ZK. Differential functional categories were identified using the Metastats method, with *p* values adjusted using the Benjamini–Hochberg false discovery rate (FDR) procedure. Features with FDR-adjusted *p* values < 0.05 were considered differentially abundant. LEfSe analysis was further used to identify differential KEGG orthologs, with *p* < 0.05 and LDA score >2.0 used as the significance thresholds for functional features (Kanehisa and Goto, 2000; Kanehisa et al., 2014).

### 2.7 Annotation and differential analysis of antibiotic resistance genes

Antibiotic resistance genes (ARGs) in the non-redundant gene catalog were annotated using the Comprehensive Antibiotic Resistance Database (CARD; version 3.1.0, downloaded on 12 March 2021) and the Antibiotic Resistance Ontology (ARO; version 4.0, downloaded on 11 March 2021). Information from ARO, including ARO accession, gene name, antibiotic class, resistance mechanism and AMR gene family, was obtained for each annotated ARG. ARGs were identified with identity >95% and e-value ≤ 1e^−5^. ARG abundance was normalized according to gene length and sequencing depth. Venn diagrams were used to analyse shared and treatment-specific ARG distributions among treatments. Relative abundance bar plots, clustering heatmaps and Circos plots were used to visualize the composition and distribution patterns of major ARO types across samples. LEfSe analysis was further performed to identify significantly enriched ARGs among treatments, with *p* < 0.05 and LDA score >2.0 used as the significance thresholds. The antibiotic classes and resistance mechanisms of candidate differential ARGs were summarized based on ARO annotation information (Cantarel et al., 2009).

### 2.8 Redundancy analysis between soil environmental factors and dominant bacterial genera

To analyze the potential associations between soil environmental factors and the community structure of dominant bacterial genera, redundancy analysis (RDA) was performed based on the abundance data of the Top 40 dominant bacterial genera and soil physicochemical and enzyme activity variables. The environmental variables included in the analysis were SOM, ${\text{NH}}_{4}^{\text{+}}$-N, A-P, SAL, SUC and CAT. Before RDA, bacterial genus abundance data were Hellinger-transformed, and environmental variables were standardized. RDA was performed using the vegan package in R, and permutation tests were used to assess the significance of the overall model, ordination axes and individual environmental variables. The RDA ordination plot was used to visualize associations between environmental factors and dominant bacterial genera (Ji et al., 2025).

### 2.9 Statistical analysis and visualization

All statistical analyses and visualizations were performed using R (R Core Team). Soil physicochemical properties and enzyme activities were expressed as mean ± standard deviation. Two-way ANOVA was used to examine variation associated with cultivation setting, cultivation treatment and their interaction. Because protected and open-field settings were not replicated across multiple independent sites, the two-way ANOVA results were interpreted as exploratory comparisons rather than fully replicated tests of independent environmental effects. Unless otherwise stated, *p* < 0.05 was considered statistically significant. Community analyses were conducted using the vegan package v2.6.4. Bray–Curtis dissimilarities were calculated from genus-level relative abundance data using vegdist; PCoA was performed using cmdscale; PERMANOVA was conducted using adonis2 with 999 permutations; and RDA was performed using rda, with significance of the overall model, canonical axes and environmental variables assessed by anova.cca with 999 permutations.

Differential abundance analyses of microbial taxa and KEGG functional categories in pairwise comparisons (NT vs. NK and ZT vs. ZK) were performed using the Metastats method. *p* values from Metastats analyses were adjusted using the Benjamini–Hochberg false discovery rate (FDR) procedure, and features with FDR-adjusted *p* values < 0.05 were considered significantly different. LEfSe analysis was used to identify microbial biomarkers, KEGG orthologs and ARGs using the Kruskal–Wallis test followed by linear discriminant analysis. The thresholds were *p* < 0.05 and LDA score >3.0 for microbial taxa, and *p* < 0.05 and LDA score >2.0 for KEGG orthologs and ARGs. Figures were generated mainly using ggplot2 for bar plots, box plots, bubble plots and ordination plots; pheatmap for heatmaps; circlize with chordDiagram for Circos plots; and ggVennDiagram or VennDiagram v1.7.3 for Venn diagrams.

## 3 Results

### 3.1 Effects of protected and open-field *Stropharia rugosoannulata* cultivation on soil physicochemical properties and enzyme activities

Compared with the corresponding uncultivated soils, *S. rugosoannulata* cultivation showed more evident effects on soil physicochemical properties and enzyme activities under protected conditions than under open-field conditions (Figure 1). Under protected conditions, NT significantly increased soil ${\text{NH}}_{4}^{\text{+}}$-N content from 52.21 mg kg^−1^ in NK to 76.67 mg kg^−1^, corresponding to an increase of 46.9%. Soil salinity and EC were significantly decreased from 1.30 mg g^−1^ and 1.11 mS cm^−1^ in NK to 0.94 mg g^−1^ and 0.80 mS cm^−1^ in NT, respectively, both representing decreases of 27.8%. For enzyme activities, SUC was significantly higher in NT than in NK, increasing from 30.46 to 38.05 mg d^−1^ g^−1^. In contrast, SOM, ${\text{NO}}_{3^{\text{-}}}$–N, A-P, A-K, pH and CAT did not differ significantly between NT and NK.

**Figure 1:**
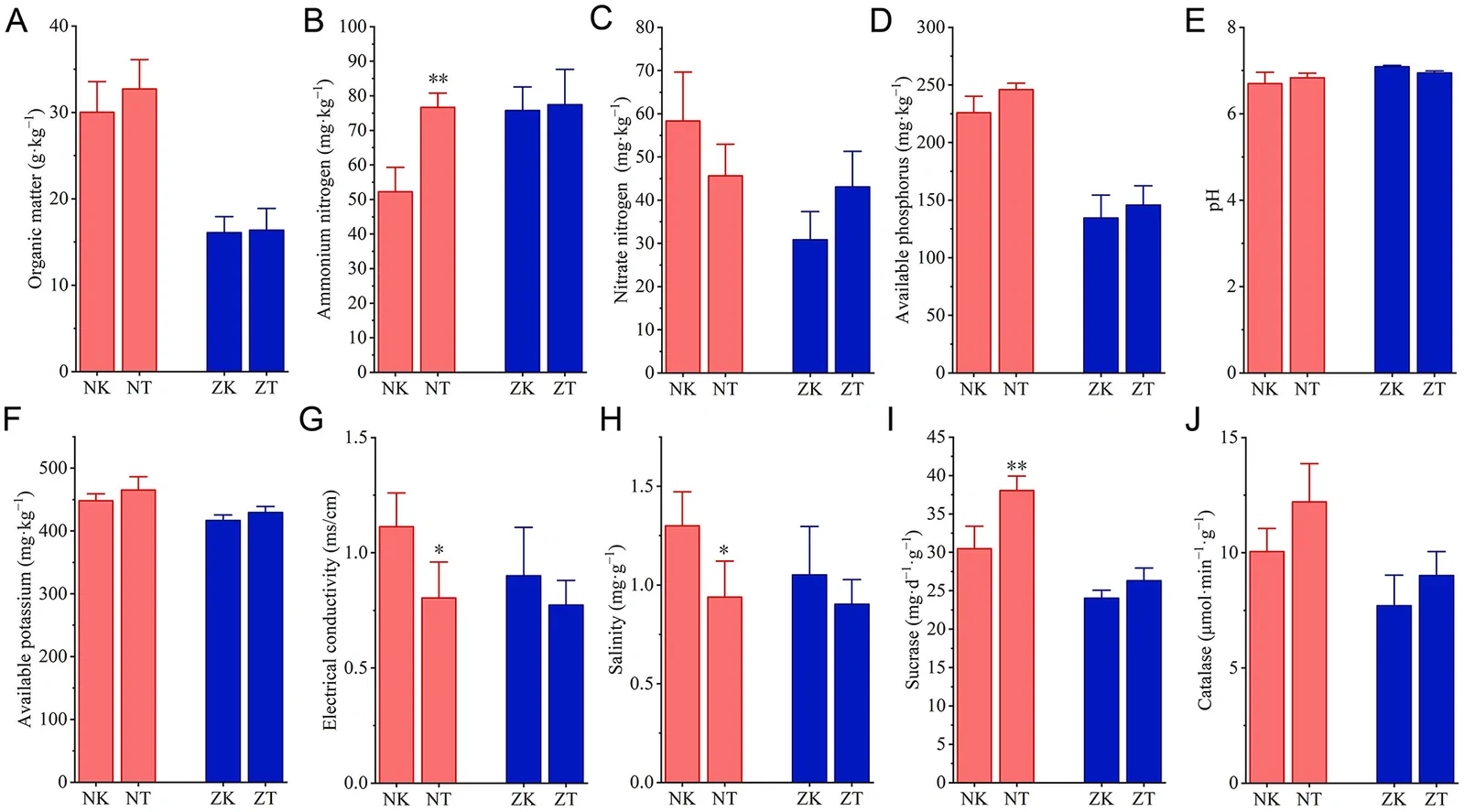
Effects of protected and open-field cultivation of *Stropharia rugosoannulata* on soil physicochemical properties and enzyme activities. **(A)** Organic matter; **(B)** ammonium nitrogen; **(C)** nitrate nitrogen; **(D)** available phosphorus; **(E)** pH; **(F)** available potassium; **(G)** electrical conductivity; **(H)** salinity; **(I)** sucrase activity; and **(J)** catalase activity. Asterisks indicate significant differences: *p* < 0.05; *p* < 0.01.

Under open-field conditions, no significant differences were detected in the measured soil physicochemical properties or enzyme activities between ZT and ZK (Figure 1). Although some variables showed numerical differences between the two treatments, these differences were not statistically significant and were therefore not interpreted as cultivation-induced changes.

Two-way ANOVA was further used as an exploratory analysis to examine variation associated with cultivation setting, cultivation treatment and their interaction (Table S1). Several soil variables differed between the protected and open-field settings, including SOM, ${\text{NH}}_{4}^{\text{+}}$-N, ${\text{NO}}_{3}^{-}$–N, A-P, A-K, SUC and CAT (*p* < 0.05). However, because protected and open-field settings were not replicated across multiple independent sites, these differences should be interpreted as setting-associated variation rather than as fully replicated environmental effects. Cultivation treatment significantly affected ${\text{NH}}_{4}^{\text{+}}$-N, SAL, EC and SUC (*p* < 0.05), with the effect on SUC reaching a highly significant level (*p* = 0.004). The interaction term was significant for ${\text{NH}}_{4}^{\text{+}}$-N and ${\text{NO}}_{3}^{-}$-N (*p* < 0.05), indicating that cultivation-associated changes in inorganic nitrogen forms varied between the two sampled cultivation settings. The interaction effect on SUC approached significance (*p* = 0.061), whereas soil pH was not significantly affected by cultivation setting, cultivation treatment or their interaction.

Overall, the measured soil variables showed setting-associated differences between protected and open-field conditions, while *S. rugosoannulata* cultivation mainly affected ${\text{NH}}_{4}^{\text{+}}$-N, salinity, EC and SUC. The significant interaction effects for ${\text{NH}}_{4}^{\text{+}}$-N and ${\text{NO}}_{3}^{-}$-N suggest that cultivation-associated changes in inorganic nitrogen forms differed between the two sampled cultivation settings, but these patterns should be interpreted within the constraints of the present sampling design.

### 3.2 Composition and abundance distribution of the soil metagenomic gene catalog

The non-redundant gene catalog constructed from the 12 soil metagenomic samples showed clear differences in gene composition among treatments (Figure 2). The UpSet analysis indicated that the gene sets of different samples contained both shared genes and a considerable number of sample-specific genes, suggesting heterogeneity in soil metagenomic gene composition among treatments (Figure 2A). The core-pan gene rarefaction curves showed that the number of pan genes continuously increased and gradually approached a plateau with increasing sample number, whereas the number of core genes rapidly decreased and then tended to stabilize (Figure 2B). These results indicate that the non-redundant gene pool in the present samples was relatively rich, while the number of genes shared among all samples was limited.

**Figure 2:**
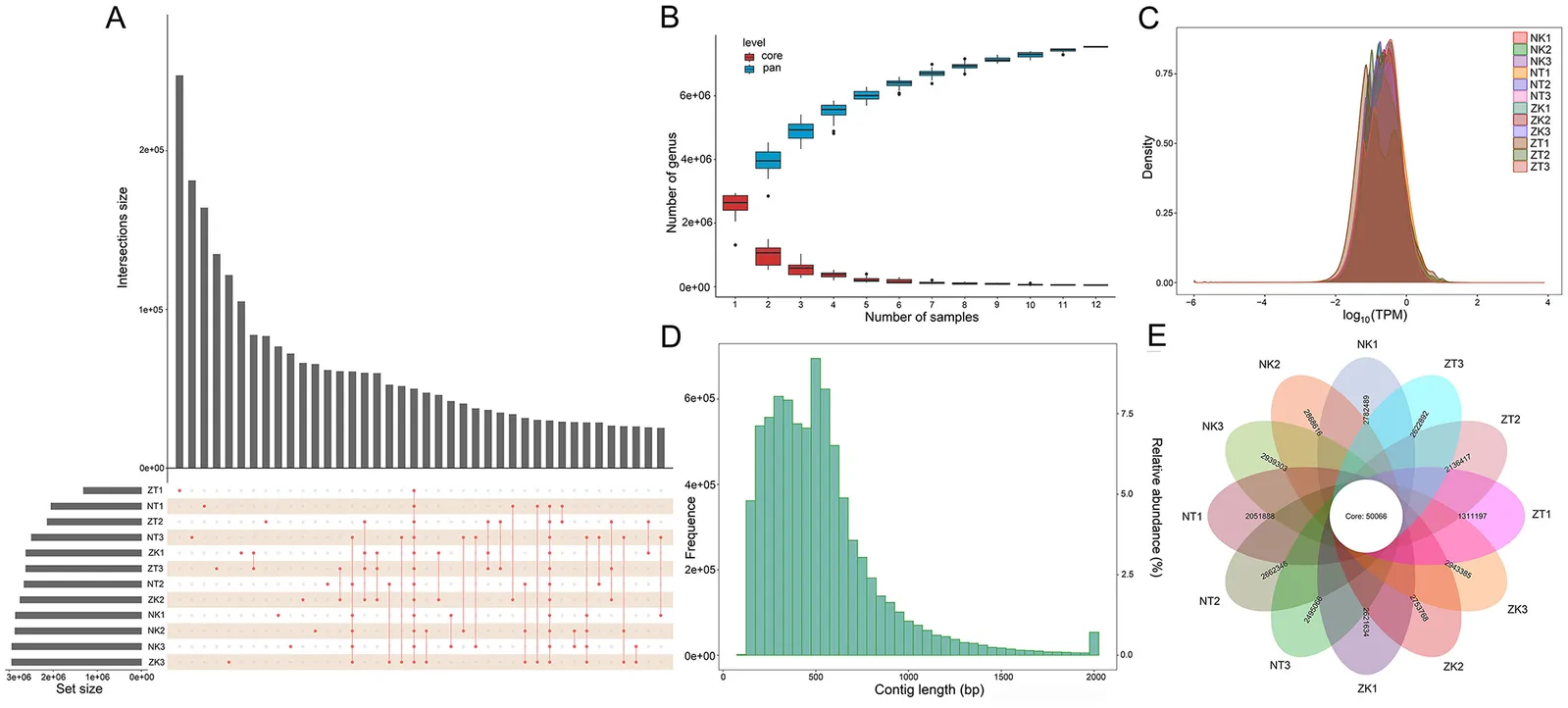
Overview of gene catalog composition and abundance distribution across soil metagenomic samples. **(**A**)** UpSet plot showing intersections of gene sets among samples. **(B)** Core-pan gene rarefaction curves. **(C)** Density distribution of log_10_-transformed TPM values. **(D)** Length distribution of contigs in the gene catalog. **(E)** Flower plot showing shared and sample-specific genes among the 12 samples.

The density distribution of normalized gene abundance based on log_10_(TPM)-transformed values showed a high degree of overlap among the 12 samples. All samples exhibited an approximately unimodal distribution, with most values concentrated between approximately −2 and 1 on the log_10_(TPM) scale, indicating good comparability of gene abundance distributions among samples (Figure 2C). The length distribution of the initially assembled contigs was right-skewed, with most contigs concentrated within approximately 100–700 bp and a relatively small proportion extending to approximately 2,000 bp (Figure 2D). The flower plot showed that the 12 samples shared 50,066 core genes (Figure 2E).

Total predicted gene numbers varied among treatment groups, with mean values of 2,863,469 ± 78,351 in NK, 2,772,929 ± 161,370 in ZK, 2,403,100 ± 314,042 in NT and 2,023,502 ± 665,256 in ZT. However, total gene number in metagenomic datasets can be strongly influenced by sequencing depth, assembly performance, contig filtering and gene prediction efficiency. Therefore, these values were used only as descriptive indicators of gene catalog size and were not interpreted as direct evidence of biological differences in microbial genetic potential among treatments. Subsequent comparisons were based mainly on normalized gene abundance distributions, taxonomic composition and functional annotation profiles.

### 3.3 Genus-level soil microbial community composition and treatment-associated taxonomic variation

At the genus level, soil microbial community composition showed treatment-associated variation in relative abundance profiles (Figure 3A). A substantial proportion of sequences could not be confidently assigned to named genera, indicating limited taxonomic resolution at the genus level in these complex soil metagenomes. Among the classified genus-level taxa, *Cellvibrio* and *Flavobacterium* were relatively abundant and showed noticeable variation among samples. Compared with the corresponding uncultivated soils, cultivated soils showed changes in the relative abundances of several classified genera. In particular, *Cellvibrio* and *Flavobacterium* showed higher relative abundances in some cultivated samples. These patterns suggest treatment-associated variation in specific classified taxa rather than dominance of these genera or broad restructuring of the overall genus-level community.

**Figure 3:**
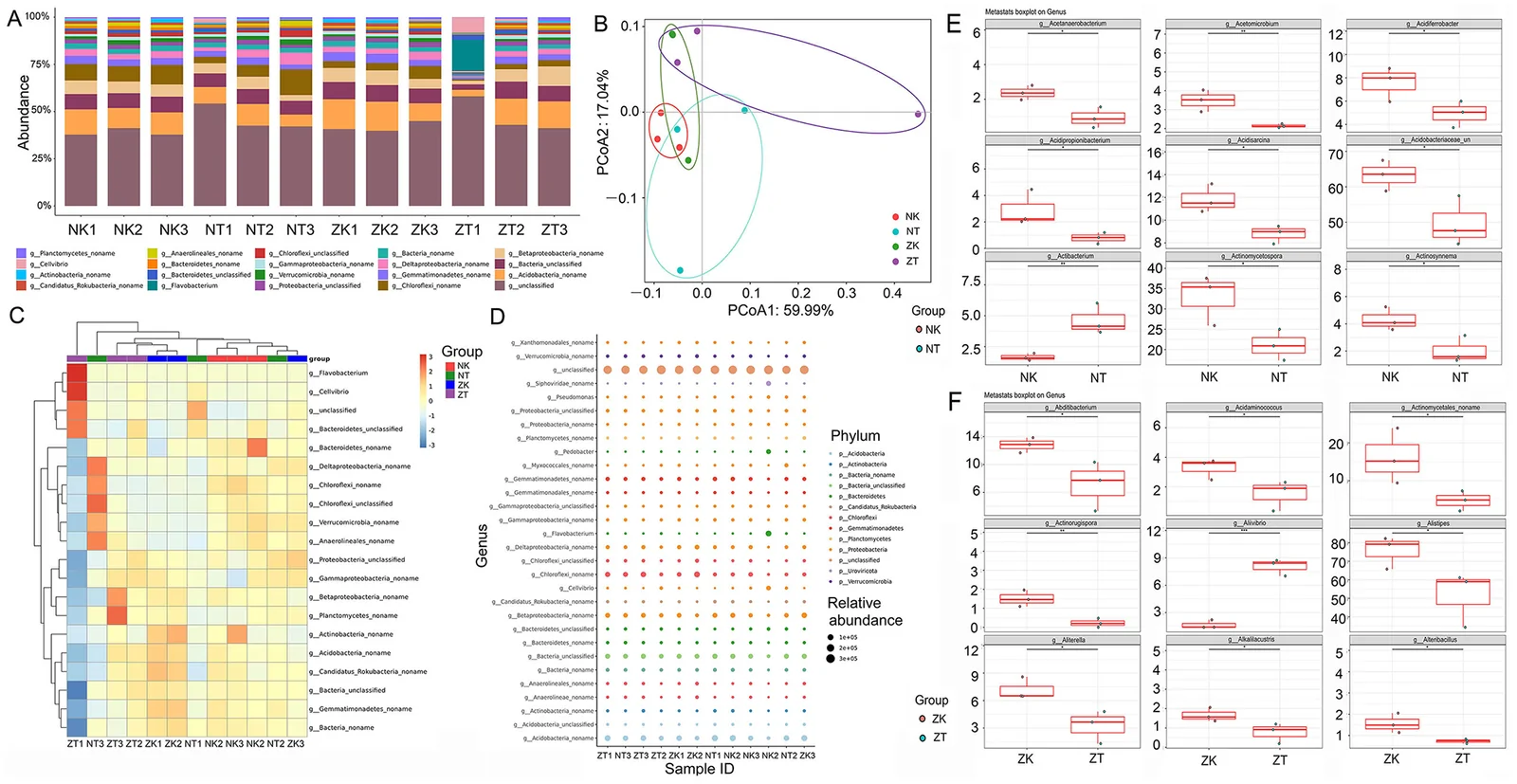
Genus-level composition and differential characteristics of soil microbial communities under different cultivation systems. **(A)** Stacked bar plot showing the relative abundance of microbial taxa at the genus level. **(B)** Principal coordinate analysis (PCoA) based on genus-level microbial community composition. **(C)** Clustering heatmap of dominant genera. **(D)** Bubble plot showing the composition of dominant genera across samples; bubble size indicates relative abundance, and color indicates phylum-level classification. **(E)** Box plots showing significantly different genera between protected cultivation soil (NT) and protected uncultivated control soil (NK). **(F)** Box plots showing significantly different genera between open-field cultivation soil (ZT) and open-field uncultivated control soil (ZK). Asterisks in the box plots indicate significant differences: ^*^p < 0.05; ^**^p < 0.01.

PCoA showed that PCoA1 and PCoA2 explained 59.99% and 17.04% of the community variation, respectively, with a cumulative explanation of 77.03% (Figure 3B). Samples from different treatments showed some distributional variation in the ordination space. Among them, ZT samples were relatively clearly separated from the other treatments along PCoA1. NK samples were more tightly clustered, whereas NT and ZK samples showed greater within-group dispersion. However, PERMANOVA based on Bray–Curtis dissimilarity indicated that the overall genus-level community structure did not differ significantly among the four treatments (*F* = 1.375, *R*^2^ = 0.340, *p* = 0.133). Therefore, the PCoA pattern should be interpreted as a visual tendency of compositional differentiation, rather than evidence of statistically significant community-level separation under the present sampling conditions.

The genus-level clustering heatmap revealed substantial sample-level heterogeneity (Figure 3C). In particular, ZT1 showed relatively high abundances of several genera, whereas ZT2 and ZT3 clustered more closely with samples from the other treatment groups. Therefore, the heatmap did not support a consistent ZT-specific clustering pattern. Genera such as *Flavobacterium* and *Cellvibrio* showed relatively high abundances in individual samples, indicating sample-level variation rather than a uniform treatment-level shift. Consistent with the PCoA result, this clustering pattern indicates sample-level variation in dominant genera but should not be interpreted as significant separation of the overall genus-level community. The bubble plot indicated that the dominant genera in different samples mainly belonged to Proteobacteria, Bacteroidetes, Gemmatimonadetes, Chloroflexi and Acidobacteria, and that the abundance distributions of major genera varied among treatments (Figure 3D).

Differential genus analysis showed that several genera differed significantly between NT and NK under protected conditions (Figure 3E). Among the displayed differential genera, *Actibacterium* was more abundant in NT, whereas *Acetanaerobacterium, Acetomicrobium, Acidiferrobacter, Acidipropionibacterium, Acidisarcina, Actinomycespora* and *Actinosynnemma* were more abundant in NK. Under open-field conditions, the differential genera between ZT and ZK mainly included *Abditibacterium, Acidaminococcus, Actinospora, Albivibrio, Alistipes, Alterella, Alkalicoccus* and *Atopobacillus* (Figure 3F). Among these genera, *Albivibrio* was more abundant in ZT, whereas the remaining differential genera were generally more abundant in ZK. These results indicate that treatment-associated microbial variation was mainly reflected in specific differential genera.

Overall, *S. rugosoannulata* cultivation was associated with changes in the relative abundances of specific genus-level taxa. Some visual differentiation was observed among individual samples in the PCoA ordination and clustering analyses; however, these patterns were not consistent across all replicates within the open-field cultivation treatment. Moreover, PERMANOVA did not detect significant differences in the overall genus-level community structure among the four treatments. Therefore, the microbial results should be interpreted primarily as treatment-associated variation in specific taxa and sample-level heterogeneity, rather than as significant restructuring of the entire genus-level microbial community.

### 3.4 LEfSe analysis of soil microbial marker taxa under different cultivation conditions

LEfSe analysis revealed multiple differential marker taxa between protected cultivation soil (NT) and protected uncultivated soil (NK) (Figures 4A, C). In NK, the enriched taxa mainly included Thaumarchaeota, Intrasporangiaceae, Pseudonocardiaceae, Chitinophagaceae, Chitinophagales, Cytophagales, Cytophagia, Sphingobacteriales and Deltaproteobacteria, all with LDA scores greater than 3.0. In contrast, fewer differential taxa were enriched in NT, mainly including Enterobacterales, Rhodobacteraceae, Bdellovibrionaceae and Myoviridae-related unnamed taxa, which also had LDA scores above 3.0. The cladogram further showed that the differential taxa enriched in NK were distributed across multiple taxonomic levels and phylogenetic branches, whereas those enriched in NT were relatively concentrated in a few specific branches. These results indicate differences in marker-taxon composition between protected cultivation soil and protected uncultivated soil.

**Figure 4:**
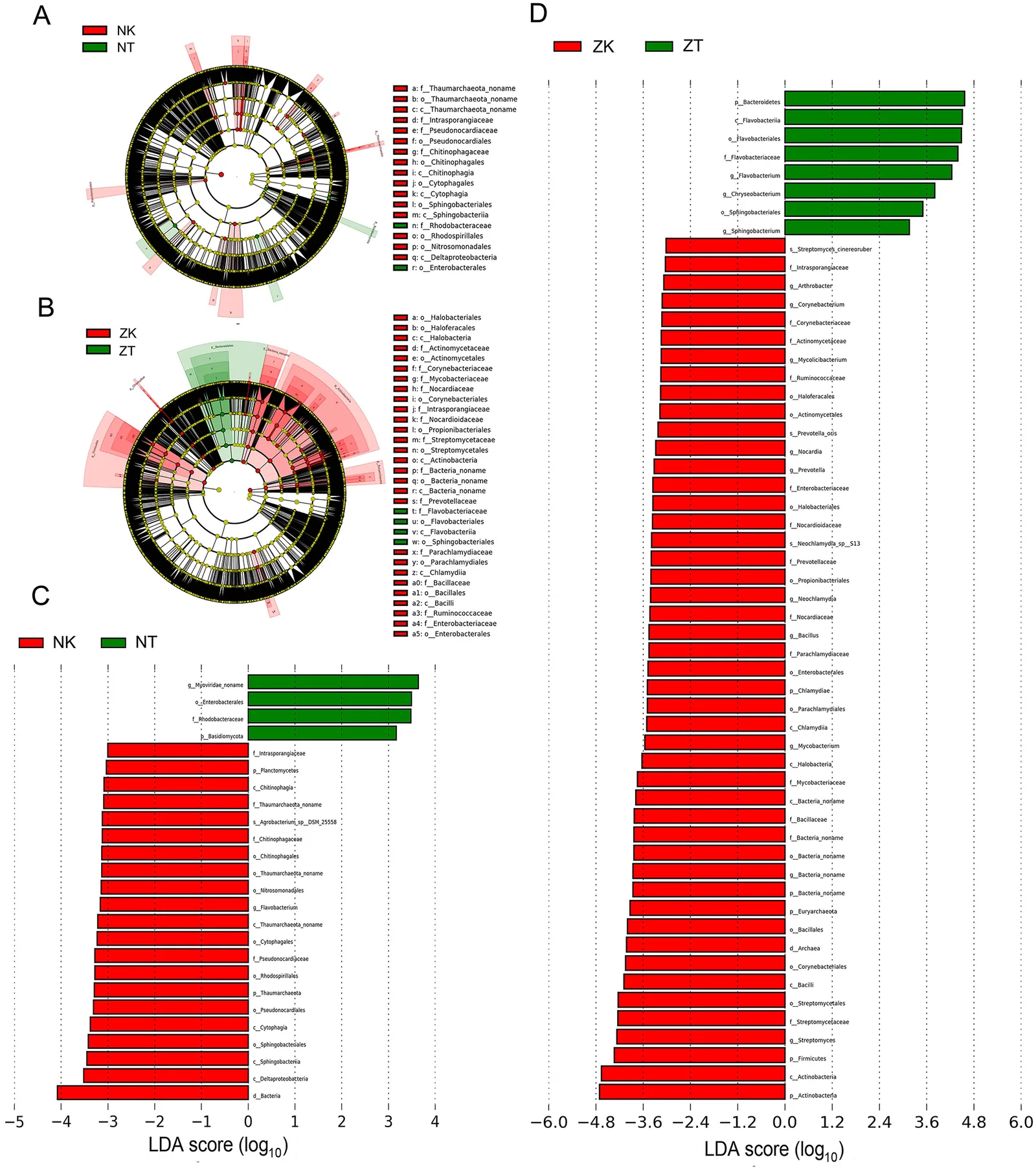
LEfSe analysis of differential microbial marker taxa under different cultivation conditions. **(A)** Cladogram of differential microbial taxa between protected cultivation soil (NT) and protected uncultivated control soil (NK). **(B)** Cladogram of differential microbial taxa between open-field cultivation soil (ZT) and open-field uncultivated control soil (ZK). **(C)** LDA score plot of differential microbial taxa between NT and NK. **(D)** LDA score plot of differential microbial taxa between ZT and ZK. Taxa labeled as “noname” or “unclassified” indicate taxonomic units that could not be further assigned to a specific taxonomic name based on database annotation. These taxa are shown only as differential marker taxa and were not used for functional interpretation.

The differential marker taxa between open-field cultivation soil (ZT) and open-field uncultivated soil (ZK) were more abundant (Figures 4B, D). The number of taxa enriched in ZK was markedly greater than that enriched in ZT. The enriched taxa in ZK mainly included Halobacteria, Halobacteriales, Halobacteriaceae, Actinobacteria, Actinomycetales, Actinomycetaceae, Corynebacteriales, Mycobacteriaceae, Nocardiaceae, Intrasporangiaceae, Streptomycetales, Bacilli, Bacillales, Clostridia, Clostridiales, Prevotellaceae, Enterobacterales and Enterobacteriaceae, with some taxa showing LDA scores approaching or exceeding 4.5. The differential taxa enriched in ZT mainly included Bacteroidetes, Flavobacteriia, Flavobacteriales, Flavobacteriaceae, *Flavobacterium*, Cytophagia, Sphingobacteriia and Sphingobacteriales, with LDA scores ranging approximately from 3.0 to 4.8.

Overall, the enrichment patterns of soil microbial marker taxa differed among cultivation conditions. Compared with protected conditions, open-field cultivation and its corresponding uncultivated soil showed a greater number of differential taxa and broader taxonomic breadth, indicating more pronounced differentiation at the marker-taxon level under open-field conditions.

### 3.5 KEGG functional characteristics of soil microbial communities under different cultivation systems

KEGG functional annotation showed that the functional composition of the 12 soil samples was generally similar at Level 2, although some differences were observed among treatments (Figure 5A). The functional categories with relatively high abundances mainly included carbohydrate metabolism, amino acid metabolism, energy metabolism, signal transduction, metabolism of cofactors and vitamins, membrane transport, replication and repair, and nucleotide metabolism. These results indicate that the functional composition of soil microbial communities was mainly associated with material metabolism, energy transformation, environmental information processing and genetic information processing.

**Figure 5:**
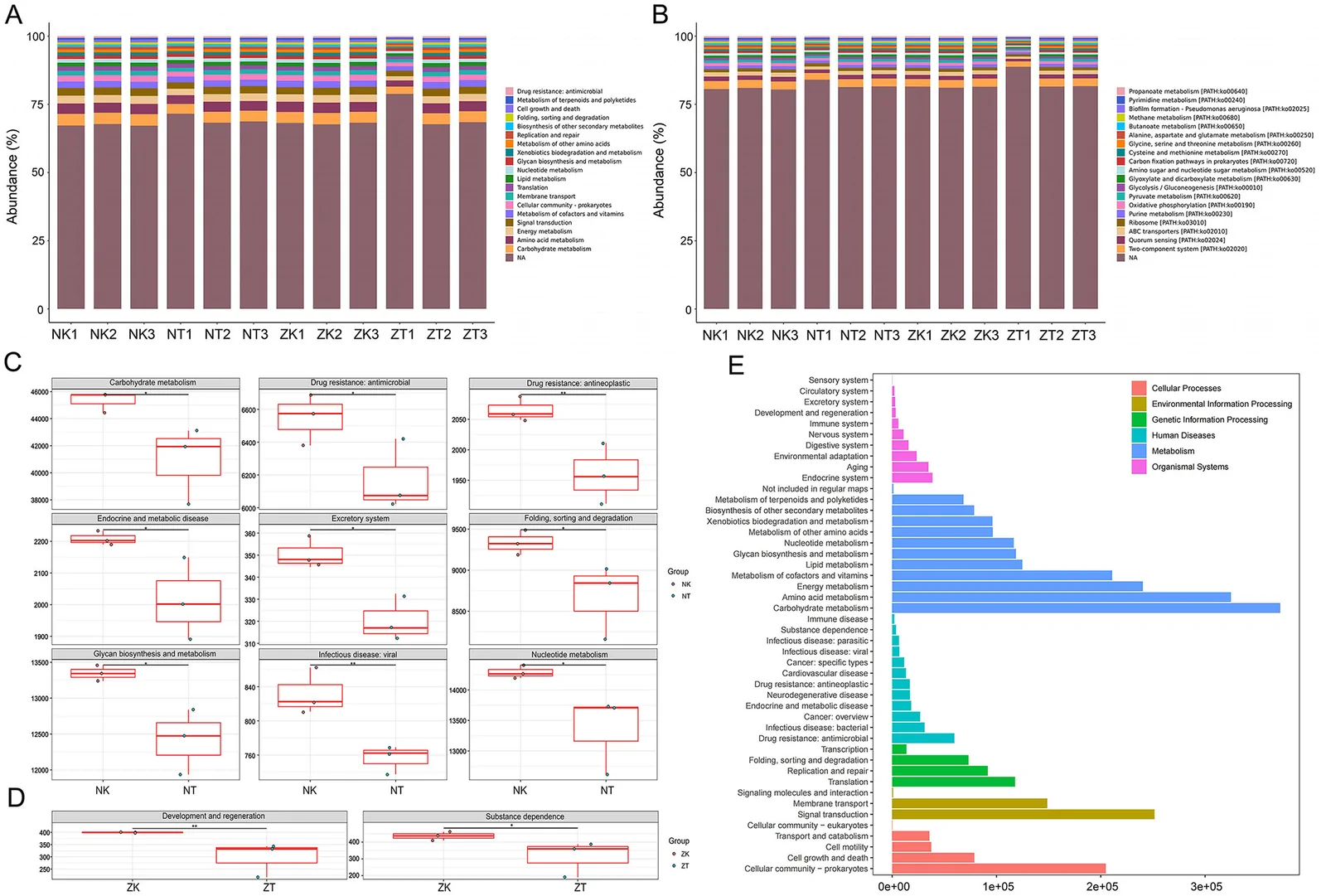
KEGG functional annotation and differential analysis of soil microbial communities under different cultivation systems. **(A)** Stacked bar plot showing KEGG functional composition at Level 2. **(B)** Stacked bar plot showing KEGG pathway-level functional composition. **(C)** Box plots of significantly different KEGG Level 2 functional categories between protected cultivation soil (NT) and protected uncultivated control soil (NK). **(D)** Box plots of significantly different KEGG Level 2 functional categories between open-field cultivation soil (ZT) and open-field uncultivated control soil (ZK). **(E)** Total number of annotations assigned to KEGG functional categories.

KEGG pathway-level annotation further showed that the functional pathway composition was relatively stable among treatments (Figure 5B). Pathways related to carbon metabolism, amino acid metabolism, energy metabolism, membrane transport, signal transduction, and metabolism of cofactors and vitamins accounted for relatively high proportions, forming the main functional background of soil microbial communities across treatments. This result suggests that, despite differences in microbial community composition under different cultivation systems, the core functional structure remained relatively stable.

Differential functional analysis revealed several significantly different KEGG Level 2 functional categories between NT and NK under protected conditions (Figure 5C). Compared with NT, NK showed higher abundances of carbohydrate metabolism, glycan biosynthesis and metabolism, nucleotide metabolism, and folding, sorting and degradation (*p* < 0.05), indicating higher annotated abundances of functions related to carbohydrate metabolism, glycan biosynthesis and metabolism, and genetic information processing in protected uncultivated soil. In addition, some resistance-related functions and disease-associated categories in the KEGG database, such as drug resistance: antimicrobial, drug resistance: antineoplastic, endocrine and metabolic disease, infectious disease: viral, and excretory system, also differed between groups. However, their ecological significance requires further interpretation based on specific functional gene annotations. Under open-field conditions, fewer KEGG Level 2 functional categories differed significantly between ZT and ZK, mainly including development and regeneration and substance dependence, both of which were more abundant in ZK (*p* < 0.05) (Figure 5D). Compared with protected conditions, the functional differences between open-field cultivation soil and its corresponding uncultivated soil were relatively limited.

The overall KEGG annotation statistics showed that carbohydrate metabolism had the highest number of annotations, followed by amino acid metabolism, energy metabolism, metabolism of cofactors and vitamins, glycan biosynthesis and metabolism, nucleotide metabolism, and membrane transport (Figure 5E). Overall, the functional composition of soil microbial communities under different cultivation systems was dominated by metabolism-related functions. Compared with open-field conditions, protected cultivation and its corresponding uncultivated soil showed more pronounced KEGG functional differences, mainly involving carbohydrate metabolism, glycan biosynthesis and metabolism, nucleotide metabolism, and protein folding, sorting and degradation.

### 3.6 Composition and differential characteristics of soil antibiotic resistance genes under different cultivation treatments

Based on the CARD/ARO annotation results, the composition of antibiotic resistance genes (ARGs) in soil samples showed both shared and treatment-specific characteristics (Figure 6A). A total of 160 ARGs were shared by all four treatment groups, indicating the presence of a relatively stable core resistome in soils under different cultivation environments. Meanwhile, each treatment contained a certain number of treatment-specific ARGs. The highest number was detected in ZT, with 64 treatment-specific ARG entries, followed by NT with 54, ZK with 50 and NK with 45. Overall, cultivated soils contained more treatment-specific ARG entries than their corresponding uncultivated soils.

**Figure 6:**
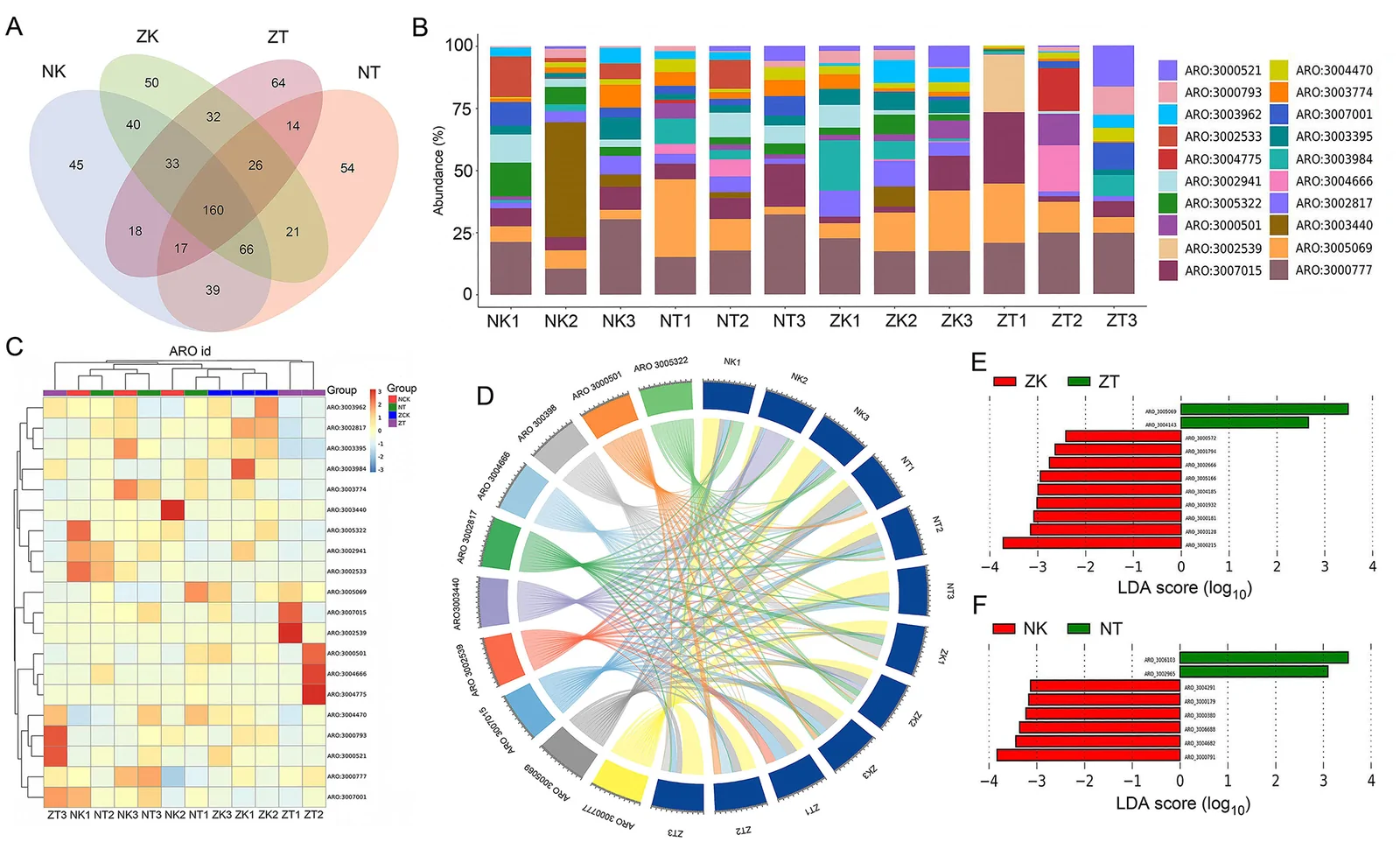
Composition, diversity and differential analysis of soil antibiotic resistance genes (ARGs) under different cultivation treatments. **(A)** Venn diagram showing shared and treatment-specific ARGs among different treatment groups. **(B)** Stacked bar plot showing the relative abundance composition of major ARO types across samples. **(C)** Clustering heatmap of ARG abundance. **(D)** Circos plot showing the correspondence between samples and major ARO types. **(E)** LDA score plot of significantly different ARGs between open-field cultivation soil (ZT) and open-field uncultivated control soil (ZK). **(F)** LDA score plot of significantly different ARGs between protected cultivation soil (NT) and protected uncultivated control soil (NK). ARGs, antibiotic resistance genes; ARO, Antibiotic Resistance Ontology.

The relative abundance composition of major ARO types varied among samples (Figure 6B). ARG composition in each sample consisted of multiple ARO entries, and some dominant AROs accounted for relatively high proportions in individual samples, indicating sample-level heterogeneity. Clustering heatmap and Circos analyses further showed that major ARGs were widely shared among treatments, while their abundance distributions varied across samples and treatment groups (Figures 6C, D). Further analysis based on ARO annotation showed that the candidate differential ARGs were mainly associated with several resistance mechanisms, including antibiotic efflux, antibiotic target alteration, antibiotic target protection or replacement, and antibiotic inactivation (Table S2). Under protected conditions, 22 candidate differential ARGs were identified between NT and NK, of which 9 were enriched in NT and 13 were enriched in NK. Representative ARGs enriched in NT included *tet(C), lsaA, gimA, vanW, OXA-917* and *mreA*, mainly associated with resistance to tetracyclines, lincosamides/streptogramins, glycopeptides and β-lactams. ARGs enriched in NK included *tet(L), tet(30), mdsC, dfrA8, bacA, mprF, ampC, aadA27* and *PDC-318*, involving multiple antibiotic classes and resistance mechanisms. Under open-field conditions, 19 candidate differential ARGs were identified between ZT and ZK, among which 18 were enriched in ZK, whereas only one ARG, *AxyX*, was enriched in ZT. Representative ARGs enriched in ZK included *tet(V), mecR1, mphC, mdsB, MexD, OXA-205, CTX-M-71, vanT, dfrA20, TriC* and *LptD*, covering resistance to tetracyclines, β-lactams, macrolides, glycopeptides, rifamycins and aminoglycosides.

LEfSe analysis further showed that the number of differentially enriched ARGs was generally higher in uncultivated soils than in the corresponding cultivated soils (Figures 6E, F). Specifically, under protected conditions, more differential ARGs were enriched in NK than in NT, and under open-field conditions, more differential ARGs were enriched in ZK than in ZT. Taken together, the Venn analysis, abundance profiles and ARO annotation results indicated that ARG composition and differential enrichment patterns varied among cultivation treatments. Cultivated soils contained more treatment-specific ARG entries, whereas uncultivated soils harbored a relatively higher number of differentially enriched ARGs.

### 3.7 Exploratory ordination of soil environmental factors and dominant bacterial genera

Redundancy analysis (RDA) was performed as an exploratory ordination to evaluate whether the measured soil environmental variables were related to the community structure of the Top 40 dominant bacterial genera. The six environmental variables included SOM, ${\text{NH}}_{4}^{\text{+}}$-N, A-P, SAL, SUC and CAT. The constrained *R*^2^ of the RDA model was 0.551, and RDA1 and RDA2 explained 40.26% and 27.82% of the constrained variation, respectively, with the first two axes together explaining 68.08% of the constrained variation (Figure 7).

**Figure 7:**
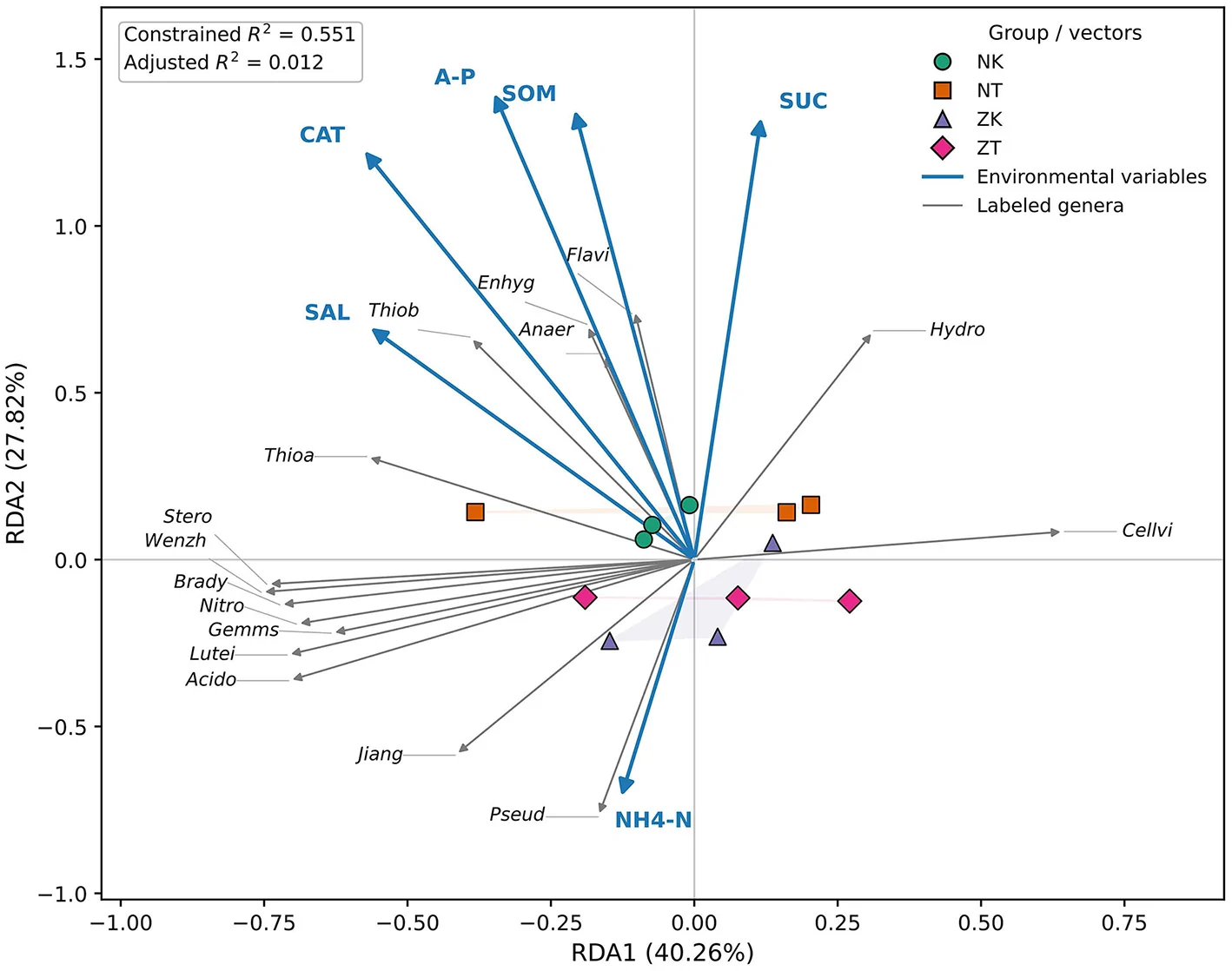
Redundancy analysis (RDA) showing associations between soil environmental factors and the community structure of the Top 40 dominant bacterial genera. Blue arrows represent environmental factors, and red labels represent dominant bacterial genera. RDA was performed based on Hellinger-transformed abundance data of the Top 40 dominant bacterial genera and standardized environmental variables. The permutation test for the overall model showed *F* = 1.023 and *p* = 0.489. Abbreviations of dominant bacterial genera are as follows: Acido, *Acidobacterium*; Pseud, *Pseudomonas*; Thiob, *Thiobacillus*; Lutei, *Luteitalea*; Wenzh, *Wenzhouxiangella*; Flavi, *Flavisolibacter*; Hydro, *Hydrogenophaga*; Stero, *Steroidobacter*; Brady, *Bradyrhizobium*; Enhyg, *Enhygromyxa*; Nitro, *Nitrospira*; Jiang, *Jiangella*; Gemms, *Gemmatimonas*; Cellvi, *Cellvibrio*; Anaer, *Anaeromyxobacter*; Thioa, *Thioalkalivibrio*.

However, permutation tests showed that the overall RDA model was not statistically significant (F = 1.023, *p* = 0.489). Marginal effect tests also showed that none of the individual environmental variables significantly explained variation in dominant bacterial genus composition (all *p* > 0.05). Therefore, the RDA results should not be interpreted as evidence of significant environmental drivers or statistically supported genus–environment relationships under the present sampling conditions.

Among the tested variables, SUC, A-P and ${\text{NH}}_{4}^{\text{+}}$-N showed relatively higher explanatory proportions, accounting for 16.60%, 13.94% and 11.12% of the variation, respectively, whereas SOM, CAT and SAL explained 6.23%, 5.38% and 5.06%, respectively. These values suggest that carbon transformation-related enzyme activity and available nutrient variables may be candidate factors for further investigation, but they should not be regarded as statistically significant explanatory effects. Accordingly, Figure 7 was used only to visualize potential multivariate patterns between soil environmental variables and dominant bacterial genera, and no specific genus–environment relationships were inferred from the ordination alone.

Overall, the RDA provided an exploratory view of possible links between soil environmental conditions and dominant bacterial genera, but did not provide statistically significant evidence that the measured environmental variables explained community variation. Future studies with larger sample sizes, multi-stage sampling and targeted correlation or network analyses are needed to test whether SUC, A-P and ${\text{NH}}_{4}^{\text{+}}$-N are consistently associated with shifts in specific bacterial taxa during *S. rugosoannulata* cultivation.

## 4 Discussion

### 4.1 Setting-associated differences in soil nutrient and enzyme responses to *Stropharia rugosoannulata* cultivation

This study showed that soil physicochemical and enzyme responses associated with S. rugosoannulata cultivation differed between the two sampled cultivation settings. Compared with protected uncultivated soil (NK), protected cultivation soil (NT) showed significantly increased ${\text{NH}}_{4}^{\text{+}}$-N content and sucrase activity, together with significantly decreased soil salinity and EC. In contrast, open-field cultivation soil (ZT) showed only non-significant trends in most variables compared with open-field uncultivated soil (ZK). These results indicate that the influence of *S. rugosoannulata* cultivation on the soil environment was not expressed as a uniform “cultivation effect,” but rather as different response patterns between the protected and open-field settings. Hao et al. (2024) reported that *S. rugosoannulata* cultivation in understory systems and rice–mushroom rotation systems, or the return of cultivation substrates to soil, could increase soil organic matter, available nutrients and some enzyme activities, but that the magnitude of these effects depended on cultivation system, soil background and management practices. This is consistent with the context-dependent response patterns observed between the two sampled settings in the present study.

Exploratory two-way ANOVA further indicated setting-associated variation in several soil nutrient and enzyme variables. SOM, ${\text{NH}}_{4}^{\text{+}}$-N, ${\text{NO}}_{3}^{-}$-N, A-P, A-K, SUC and CAT differed significantly between the protected and open-field settings, although these differences should not be interpreted as independent environmental effects because the two settings were not replicated across multiple independent sites. By contrast, cultivation treatment mainly affected ${\text{NH}}_{4}^{\text{+}}$-N, SAL, EC and SUC, while the statistical interaction term between cultivation setting and cultivation treatment was significant for ${\text{NH}}_{4}^{\text{+}}$-N and ${\text{NO}}_{3}^{-}$-N. These interaction patterns suggest that cultivation-associated changes in inorganic nitrogen forms differed between the two sampled settings; however, they do not establish an independent environmental interaction effect because setting and background soil conditions could not be fully separated in the present design. Soil inorganic nitrogen forms are generally regulated by organic matter decomposition, microbial mineralization, nitrification, and plant–microbe competition for nitrogen. However, the enzymatic mechanisms underlying these nitrogen transformations should be interpreted cautiously in the present study, because only sucrase and catalase activities were measured, whereas key enzymes directly involved in nitrogen and phosphorus transformation, such as urease, protease and phosphatase, were not assayed. The significant increase in ${\text{NH}}_{4}^{\text{+}}$-N in NT may reflect cultivation-associated changes in nitrogen availability related to straw substrate decomposition and local soil–substrate conditions. Kuzyakov and Xu (2013) indicated that strong competition and turnover processes occur among plants, microorganisms and soil nitrogen pools, and that these processes are highly sensitive to moisture, temperature and resource availability. Therefore, the distinct ${\text{NH}}_{4}^{\text{+}}$-N response observed in the protected setting may be related to differences in substrate supply, moisture conditions and other local environmental characteristics, although the relative contribution of these factors cannot be separated in the present study.

The significant increase in sucrase activity was another important feature of the soil response under protected cultivation. Soil enzymes are key functional indicators linking organic matter decomposition, microbial metabolism and nutrient release, and they can sensitively reflect soil carbon, nitrogen and phosphorus cycling processes. Ecoenzymatic stoichiometry studies have shown that soil enzyme activities are closely associated with microbial resource acquisition and organic nutrient transformation (Yang Y. et al., 2020). Reviews of soil enzymes have also emphasized that enzyme activities are highly responsive to organic substrate input, environmental conditions and changes in microbial activity (Sinsabaugh et al., 2009). In this study, SUC mainly reflected carbohydrate decomposition and soil organic carbon transformation, whereas CAT reflected oxidative stress response and microbial metabolic activity. Therefore, the enzyme activity results provide information on C transformation-related activity and oxidative response, but do not directly explain N- or P-cycling enzyme processes. In the present study, the significant increase in SUC in NT suggests that carbohydrate decomposition-related enzyme activity was higher in cultivated soil than in the corresponding uncultivated soil under the protected setting. This result is consistent with the findings of Hao et al. (2024), who observed that the return of *S. rugosoannulata* cultivation substrate in a rice–mushroom rotation system increased sucrase, amylase and laccase activities. These findings suggest that mycelial activity and organic substrate input associated with *S. rugosoannulata* cultivation may contribute to changes in soil carbon transformation-related enzyme activity, although direct carbon turnover rates were not measured in the present study.

Notably, soil salinity and EC were significantly lower in NT than in NK. This result differs from the salinity accumulation commonly reported in long-term protected agriculture, where high water and fertilizer inputs, limited rainfall leaching and relatively closed environmental conditions often promote soil salinization, acidification and nutrient imbalance. However, the protected *S. rugosoannulata* cultivation system in this study differed from conventional high-input protected vegetable systems. The cultivation bed was covered with straw-based substrate, and during the fruiting period, water was sprayed twice per week for approximately 5 min each time to maintain moisture for mushroom production. Therefore, compared with the protected uncultivated soil, the cultivated soil was affected not only by the protected setting, but also by substrate coverage and periodic water input at the soil–substrate interface. A plausible explanation for the lower salinity and EC in NT is that straw coverage may have reduced surface evaporation and upward capillary salt accumulation, while repeated water spraying may have contributed to dilution or downward redistribution of soluble salts within the sampled soil layer. Previous studies have shown that straw mulching can reduce soil evaporation, increase soil water retention and suppress salt accumulation in the surface or root-zone soil by limiting upward salt movement (Zhao et al., 2014). This provides a possible explanation for the lower salinity and EC observed in the soil beneath the cultivation substrate. However, because soluble ion composition, salt balance, evaporation rate and water flux were not directly measured in this study, this interpretation should be regarded as a plausible mechanism rather than direct evidence. Studies on protected soil degradation have shown that salinization and nutrient imbalance are important constraints on soil productivity, while organic matter management and crop rotation are important approaches for mitigating soil degradation (Hu et al., 2017; Yan et al., 2024). Future studies should therefore include ion composition analysis, water movement monitoring and multi-cycle observations to determine whether the lower salinity and EC observed here represent a consistent cultivation-associated response in protected *S. rugosoannulata* systems.

Overall, our results indicate that soil nutrient and enzyme responses associated with *S. rugosoannulata* cultivation differed between the protected and open-field settings. Under protected conditions, straw coverage, periodic water spraying during the fruiting period and the local soil–substrate microenvironment may have contributed to the more evident changes in ${\text{NH}}_{4}^{\text{+}}$-N, salinity, EC and sucrase activity observed in NT relative to NK. Under open-field conditions, greater exposure to rainfall, temperature fluctuations and background field heterogeneity may have contributed to the weaker detectable differences between ZT and ZK, although these mechanisms were not directly tested. These findings are also consistent with the view that soil organic matter stabilization and nutrient transformation are not determined solely by the properties of organic inputs, but are jointly regulated by environmental conditions, microbial processes and organic matter–mineral interactions (Schmidt et al., 2011; Cotrufo et al., 2013). Therefore, when evaluating the soil ecological effects of *S. rugosoannulata* cultivation, it is necessary to consider not only the cultivation practice itself, but also the background characteristics of the sampled cultivation setting and the local surface-soil microenvironment associated with straw coverage, water management and substrate decomposition, rather than attributing changes in soil nutrients and enzyme activities to cultivation treatment alone. Future studies should include N- and P-cycling enzyme activities, such as urease, protease and phosphatase, together with functional genes related to nitrogen and phosphorus transformation, to better clarify the mechanisms by which *S. rugosoannulata* cultivation affects soil inorganic nitrogen forms across different cultivation settings.

### 4.2 Differential responses of microbial community composition and functional potential to cultivation treatments

As described above, PERMANOVA did not detect significant separation in the overall genus-level community structure among treatments. Therefore, microbial responses in this study should be interpreted mainly as treatment-associated variation in specific classified genera and marker taxa, rather than as broad restructuring of the entire genus-level community. KEGG functional annotation showed that the core functional composition of soil microbial communities was generally stable across treatments, mainly including carbohydrate metabolism, amino acid metabolism, energy metabolism, membrane transport, signal transduction, metabolism of cofactors and vitamins, and nucleotide metabolism. This indicates that, despite some differences in microbial taxonomic composition, the soil microbial communities maintained a relatively stable basic metabolic functional structure. Such functional stability may be partly explained by functional redundancy among soil microorganisms. In complex soil communities, broad metabolic functions are often encoded by multiple phylogenetically distinct taxa; therefore, changes in the relative abundance of particular genera may be compensated by functionally equivalent taxa carrying similar metabolic genes. This functional compensation can buffer community-level functional potential against moderate taxonomic shifts, especially for widespread functions such as carbohydrate metabolism, amino acid metabolism and energy metabolism (Allison and Martiny, 2008; Fierer et al., 2012; Louca et al., 2016, 2018).

In the present study, the higher relative abundances of genera such as Cellvibrio and Flavobacterium in cultivated soils may indicate that specific taxa potentially associated with complex organic matter transformation were responsive to straw-based substrate input. However, the overall KEGG functional composition remained relatively stable, implying that these taxonomic shifts may have occurred within a functionally redundant microbial background. In other words, *S. rugosoannulata* cultivation may have changed the identity and relative abundance of specific relatively abundant or marker taxa without substantially altering the broad functional repertoire of the soil metagenome. This pattern is more consistent with functional compensation among taxa than with complete functional restructuring of the microbial community.

Under protected conditions, more significantly different KEGG Level 2 functional categories were detected between NT and NK, mainly involving carbohydrate metabolism, glycan biosynthesis and metabolism, nucleotide metabolism, and protein folding, sorting and degradation. These functional categories have clear ecological relevance in straw-based mushroom cultivation systems. Carbohydrate metabolism and glycan biosynthesis/metabolism are closely related to the transformation of plant- and fungal-derived polysaccharides, including cellulose, hemicellulose, starch-like compounds, fungal cell-wall components and extracellular polymeric substances. Therefore, differences in these categories may indicate a redistribution of microbial functional potential associated with organic substrate processing and soil carbon transformation. Notably, the relative abundances of these KEGG Level 2 categories were higher in NK than in NT, which should not be interpreted as stronger carbohydrate decomposition in NK. KEGG profiles derived from metagenomic data reflect the relative abundance of annotated functional genes, rather than gene expression, enzyme activity or actual decomposition rates (Prosser, 2015). Therefore, the lower relative proportion of carbohydrate metabolism and glycan biosynthesis/metabolism in NT does not necessarily indicate reduced carbon turnover under protected cultivation. This interpretation is also consistent with the significant increase in sucrase activity under protected cultivation, although KEGG annotation alone cannot confirm actual carbon turnover rates.

The differences in nucleotide metabolism and protein folding, sorting and degradation may reflect changes in microbial resource allocation related to growth, DNA/RNA synthesis, protein turnover and stress adaptation under the relatively humid, substrate-rich soil–substrate microenvironment created by protected cultivation. However, because metagenomic data represent functional potential rather than gene expression or process rates, these KEGG differences should not be interpreted as direct evidence of enhanced metabolic activity. The apparently higher relative abundance of these broad functional categories in NK may instead reflect a different background heterotrophic gene pool or a shift in the proportional allocation of annotated genes, rather than a higher level of microbial activity. In addition, straw decomposition in *S. rugosoannulata* cultivation occurs mainly in the straw-based substrate and fungal mycelial zone, whereas the metagenomic analysis in this study was based on soil collected beneath the cultivation substrate after cultivation. Thus, substrate decomposition processes and soil bacterial functional gene profiles may not be fully synchronized. Future studies combining metatranscriptomics, enzyme assays and carbon turnover measurements are needed to determine whether these differences translate into changes in actual microbial activity and soil carbon transformation rates.

RDA results showed that SUC, A-P and ${\text{NH}}_{4}^{\text{+}}$-N had relatively higher explanatory proportions for the composition of the Top 40 dominant bacterial genera. However, neither the overall RDA model nor the permutation tests for individual environmental factors reached statistical significance. Therefore, these results should be interpreted only as exploratory ordination patterns rather than evidence of significant environmental drivers. The relatively higher explanatory proportions of SUC, A-P and ${\text{NH}}_{4}^{\text{+}}$-N may help identify candidate variables for future investigation, but they do not provide statistically supported genus–environment relationships in the present dataset.

Overall, *S. rugosoannulata* cultivation was associated with treatment-specific variation in dominant taxa, marker taxa and selected functional categories, rather than significant restructuring of the overall community. The apparent stability of broad KEGG functional profiles may be related to functional redundancy and compensation among soil microbial taxa, whereas the differences in carbohydrate metabolism, glycan biosynthesis, nucleotide metabolism and protein processing under protected conditions suggest a possible shift in functional potential related to substrate processing and microbial resource allocation. These responses may be associated with sampling stage, straw-derived local microenvironments, high moisture conditions caused by regular irrigation and background soil heterogeneity. Because soil O_2_ concentration, CO_2_ emission rate, redox potential, soil respiration intensity and gene expression were not directly measured, these interpretations should be regarded as moderate inferences based on cultivation mode and microbial functional potential. Future studies combining multi-stage sampling, soil gas process monitoring, functional gene expression analysis and process-level measurements are needed to verify whether the observed functional differences correspond to actual changes in carbon turnover and microbial activity.

### 4.3 Treatment-specific changes in the soil resistome and their implications for ecological risk indication

Antibiotic resistance genes (ARGs) are important components of the soil microbial gene pool, and their occurrence does not necessarily depend on anthropogenic antibiotic inputs. Soils and other natural environments are widely recognized as important reservoirs of ARGs, and some environmental ARGs are genetically related to resistance genes found in clinical pathogens, suggesting that environmental resistomes may serve as potential sources for the evolution and dissemination of antibiotic resistance (Allen et al., 2010; Forsberg et al., 2012). However, because the ARG data in this study were derived from metagenomic annotation, the following discussion focuses mainly on ARG occurrence, relative abundance patterns and treatment-associated differences, rather than providing a broad assessment of environmental resistome risk. In this study, ARGs in soil metagenomes were annotated using the CARD/ARO database, and 160 ARGs were shared across the four treatment groups. This indicates the presence of a relatively stable core resistome in both protected and open-field soils, consistent with the view that ARGs are widely distributed in natural environments and may represent part of the genetic composition formed during the long-term evolution and environmental adaptation of soil microbial communities (Forsberg et al., 2012).

ARG composition showed clear treatment-specific patterns. The numbers of treatment-specific ARG entries in ZT and NT were higher than those in their corresponding uncultivated soils, suggesting that *S. rugosoannulata* cultivation may alter the detectable ARG profile in soil. However, a higher number of ARG entries should not be directly interpreted as increased ecological risk. Venn analysis mainly reflects ARG occurrence, whereas LEfSe emphasizes abundance differences and effect sizes. In this study, cultivated soils contained more treatment-specific ARG entries, whereas uncultivated soils harbored more differentially enriched ARGs, indicating that ARG occurrence specificity and abundance enrichment were not fully consistent. Therefore, the effects of *S. rugosoannulata* cultivation on the soil resistome should be evaluated by jointly considering ARG number, abundance, resistance mechanism, potential host, mobile genetic elements and environmental selection pressures, rather than inferring resistance risk solely from treatment-specific ARG richness (Larsson and Flach, 2022).

The candidate differential ARGs identified in this study were mainly associated with antibiotic efflux, target alteration, target protection or replacement, and antibiotic inactivation, covering multiple resistance classes, including tetracyclines, β-lactams, macrolides, glycopeptides, rifamycins and aminoglycosides. These results indicate that ARG variation among treatments involved multiple resistance mechanisms, but they should be interpreted as differences in annotated resistome composition rather than direct evidence of changes in resistance activity or transmission risk. Efflux pump-related genes and target protection or alteration genes are commonly detected in environmental samples, and their broad distribution may be associated with soil microbial diversity, natural antibiotic-producing microorganisms, environmental stress adaptation and multidrug resistance systems. Large-scale metagenomic studies have shown that ARG composition differs markedly among habitats, with soils, waters, sediments and agricultural systems forming distinct resistome profiles; in agricultural environments, ARG abundance and composition may also be shaped by organic inputs, metal elements, microbial community structure and management practices (Nesme et al., 2014).

From the perspective of soil health and continuous edible mushroom cultivation, shifts in ARG profiles may provide useful supplementary indicators of soil microbial ecological change. Continuous cropping obstacles in *S. rugosoannulata* production are unlikely to result from a single factor, but may involve substrate residues, altered nutrient availability, salinity changes, accumulation of pathogenic or opportunistic microorganisms, and imbalance in microbial functions. This is consistent with the view that continuous cropping obstacles are generally associated with combined changes in soil microbial communities, nutrient availability and soil ecological functions (Zeeshan Ul Haq et al., 2023). In the present study, *S. rugosoannulata* cultivation not only altered soil ${\text{NH}}_{4}^{\text{+}}$-N, salinity, EC and sucrase activity, but also affected microbial marker taxa, functional categories and ARG composition. Therefore, treatment-specific ARG variation may reflect soil microbial ecological succession driven by cultivation substrate input, soil environmental changes and microbial community reorganization, rather than serving as direct causal evidence for continuous cropping obstacles.

Under protected conditions, 22 candidate differential ARGs were identified between NT and NK, whereas 19 candidate differential ARGs were detected between ZT and ZK under open-field conditions, with more ARGs enriched in ZK. These results suggest that ARG composition varied with cultivation background. In edible mushroom cultivation systems without direct antibiotic application, ARG variation is more likely to reflect the combined effects of background soil microbial communities, organic substrate input, salinity and nutrient changes, and environmental filtering, rather than enhanced antibiotic selection pressure. Therefore, the ARG results in this study should be interpreted as supplementary indicators of soil microbial ecological change rather than as direct evidence of ecological risk.

Overall, *S. rugosoannulata* cultivation altered soil ARG composition and differential enrichment patterns, but these changes should be interpreted in the broader context of soil biochemical responses and microbial community reorganization. Under protected conditions, the increase in ${\text{NH}}_{4}^{\text{+}}$-N and sucrase activity, together with the decrease in salinity and EC, suggests enhanced nitrogen availability and enzyme-mediated organic matter turnover, which may partly explain the enrichment of specific microbial taxa and the differences in functional categories related to carbohydrate metabolism, glycan biosynthesis and nucleotide metabolism. By contrast, the weaker biochemical responses under open-field conditions were accompanied by different marker taxa and ARG enrichment patterns, indicating that background environmental filtering and field heterogeneity may have played a stronger role in shaping microbial and resistome responses. From a system-level perspective, *S. rugosoannulata* cultivation did not induce a uniform shift in soil microbial ecology, but generated cultivation-setting-dependent responses involving nutrient transformation, microbial taxonomic shifts, functional stability and resistome variation. However, because the protected and open-field cultivation settings were not replicated across multiple independent sites, these patterns should be interpreted as context-dependent responses within the sampled system. Although corresponding uncultivated soils were included within each setting as local background controls, the present design cannot fully separate cultivation-setting effects from background soil heterogeneity.

It should also be emphasized that metagenomic ARG annotation mainly reflects the occurrence and abundance potential of resistance genes, but does not directly indicate ARG expression levels, host identity, mobility or actual transmission risk. Environmental antibiotic resistance risk depends on whether ARGs are located on mobile genetic elements, whether they are carried by potential pathogens, whether they are subject to sustained selection pressure, and whether transmission pathways exist from environmental microbial communities to human- or animal-associated microbiota (Larsson and Flach, 2022). Future studies should combine qPCR-based quantification, metatranscriptomic analysis, identification of mobile genetic elements, ARG host tracking and multi-season continuous sampling to further evaluate the long-term effects of *S. rugosoannulata* cultivation on soil resistome stability and transmission potential.

## 5 Conclusions

This study shows that the soil ecological responses associated with *Stropharia rugosoannulata* cultivation varied between the two sampled cultivation settings. Protected cultivation produced more pronounced changes in soil biochemical properties than open-field cultivation, particularly in inorganic nitrogen status, soil salinity/EC and sucrase activity. These findings indicate that cultivation setting was associated with differences in soil nutrient status and biochemical responses during edible mushroom production, although protected/open-field differences should be interpreted within the constraints of the present sampling design. Metagenomic analyses further showed that *S. rugosoannulata* cultivation was associated with variation in specific microbial taxa and functional gene profiles, without significant restructuring of the overall genus-level microbial community. The higher relative abundances of lignocellulose-associated genera such as *Cellvibrio* and *Flavobacterium* suggests that substrate-derived organic inputs play an important role in shaping soil microbial ecological responses. Although core microbial functions remained relatively stable, cultivation environment affected the extent of taxonomic and functional differentiation, while ARG profiles exhibited treatment-specific patterns. Collectively, these results provide metagenomic evidence that the impacts of edible mushroom cultivation on soil biochemical processes, microbial communities, and resistome composition are environment-dependent rather than universal. Our findings highlight the importance of considering cultivation environment and substrate-induced surface soil microenvironments when evaluating soil health, ecological sustainability, and potential environmental risks in continuous mushroom cultivation systems.

## Data Availability

The original contributions presented in the study are included in the article/Supplementary material, further inquiries can be directed to the corresponding authors.
