# Supplementary material for: Environment-dependent responses of soil physicochemical properties, microbial communities and functional gene profiles to protected and open-field cultivation of Stropharia rugosoannulata
Source: Front Microbiol. 2026 Oct 14;17:1912245. doi: 10.3389/fmicb.2026.1912245 (PMC13713400; doi:10.3389/fmicb.2026.1912245)
Supplement: Supplementary file 1 [file Table_1.docx]

**Table S1.** Results of two-way ANOVA for soil physicochemical properties and enzyme activities.

| Variable | Environmental condition F (*p*) | Cultivation treatment F (*p*) | Environmental condition × cultivation treatment F (*p*) |
| --- | --- | --- | --- |
| SOM | 59.27（*p* < 0.001） | 0.59（*p* = 0.466） | 0.38（*p* = 0.556） |
| NH_4_⁺-N | 9.48（*p* = 0.015） | 10.92（*p* = 0.011） | 8.32（*p* = 0.020） |
| NO_3_⁻-N | 9.21（*p* = 0.016） | 0.00（*p* = 0.963） | 6.32（*p* = 0.036） |
| pH | 3.85（*p* = 0.085） | 0.00（*p* = 0.960） | 1.12（*p* = 0.321） |
| A-P | 94.93（*p* < 0.001） | 2.51（*p* = 0.152） | 0.20（*p* = 0.664） |
| A-K | 17.45（*p* = 0.003） | 3.50（*p* = 0.098） | 0.07（*p* = 0.802） |
| SAL | 1.88（*p* = 0.208） | 6.05（*p* = 0.039） | 1.07（*p* = 0.332） |
| EC | 1.88（*p* = 0.208） | 6.05（*p* = 0.039） | 1.07（*p* = 0.332） |
| SUC | 56.34（*p* < 0.001） | 16.65（*p* = 0.004） | 4.77（*p* = 0.061） |
| CAT | 12.48（*p* = 0.008） | 4.85（*p* = 0.059） | 0.29（*p* = 0.604） |

**Table S2.** ARO annotations of candidate differential antibiotic resistance genes under different cultivation treatments.

| **Comparison** | **ARO ID** | **ARO name** | **Drug class** | **Resistance mechanism** | **AMR gene family** | **Enriched group** | **Mean abundance in control** | **log2FC (treatment/control)** | **BH-FDR q** | **Mann-Whitney P** |
| --- | --- | --- | --- | --- | --- | --- | --- | --- | --- | --- |
| NT vs NK | ARO:3000167 | tet(C) | tetracycline antibiotic | antibiotic efflux | major facilitator superfamily (MFS) antibiotic efflux pump | NT | 0.9855 | 19.9104 | 0.5285 | 0.0636 |
| NT vs NK | ARO:3000179 | tet(L) | tetracycline antibiotic | antibiotic efflux | major facilitator superfamily (MFS) antibiotic efflux pump | NK | 0.2922 | -18.1568 | 0.5285 | 0.0636 |
| NT vs NK | ARO:3000300 | lsaA | lincosamide antibiotic; streptogramin antibiotic; streptogramin A antibiotic; streptogramin B antibiotic; pleuromutilin antibiotic | antibiotic target protection | lsa-type ABC-F protein | NT | 1.7456 | 20.7353 | 0.5285 | 0.0636 |
| NT vs NK | ARO:3000463 | gimA | macrolide antibiotic | antibiotic inactivation | gimA family macrolide glycosyltransferase | NT | 0.8071 | 2.1532 | 0.5285 | 0.1000 |
| NT vs NK | ARO:3000561 | tet(30) | tetracycline antibiotic | antibiotic efflux | major facilitator superfamily (MFS) antibiotic efflux pump | NK | 0.6543 | -19.3195 | 0.5285 | 0.0636 |
| NT vs NK | ARO:3000791 | mdsC | monobactam; carbapenem; cephalosporin; cephamycin; penam; phenicol antibiotic; penem | antibiotic efflux | resistance-nodulation-cell division (RND) antibiotic efflux pump | NK | 2.0043 | -20.9347 | 0.5285 | 0.0636 |
| NT vs NK | ARO:3001313 | facT | elfamycin antibiotic | antibiotic efflux | major facilitator superfamily (MFS) antibiotic efflux pump | NK | 1.7256 | -2.5961 | 0.5285 | 0.1000 |
| NT vs NK | ARO:3002518 | OCH-5 | monobactam; cephalosporin; cephamycin; penam; penem | antibiotic inactivation | OCH beta-lactamase | NT | 1.9741 | 20.9128 | 0.5285 | 0.0636 |
| NT vs NK | ARO:3002813 | lmrB | lincosamide antibiotic | antibiotic efflux | ATP-binding cassette (ABC) antibiotic efflux pump | NT | 0.6709 | 5.6787 | 0.5285 | 0.0765 |
| NT vs NK | ARO:3002827 | tlrC | macrolide antibiotic; lincosamide antibiotic | antibiotic target protection | Miscellaneous ABC-F subfamily ATP-binding cassette ribosomal protection proteins | NK | 1.9642 | -3.9487 | 0.5285 | 0.0765 |
| NT vs NK | ARO:3002863 | dfrA8 | diaminopyrimidine antibiotic | antibiotic target replacement | trimethoprim resistant dihydrofolate reductase dfr | NK | 0.4528 | -2.8457 | 0.5285 | 0.0765 |
| NT vs NK | ARO:3002965 | vanW gene in vanG cluster | glycopeptide antibiotic | antibiotic target alteration | vanW; glycopeptide resistance gene cluster | NT | 0.2639 | 18.0094 | 0.5285 | 0.0636 |
| NT vs NK | ARO:3002986 | bacA | peptide antibiotic | antibiotic target alteration | undecaprenyl pyrophosphate related proteins | NK | 1.1513 | -3.2948 | 0.5285 | 0.0765 |
| NT vs NK | ARO:3003324 | Bacillus subtilis mprF | peptide antibiotic | antibiotic target alteration | defensin resistant mprF | NK | 0.5124 | -18.9668 | 0.5285 | 0.0636 |
| NT vs NK | ARO:3003395 | Mycobacterium tuberculosis rpsL mutations conferring resistance to Streptomycin | aminoglycoside antibiotic | antibiotic target alteration | antibiotic-resistant rpsL | NK | 2.1061 | -0.7540 | 0.5285 | 0.1000 |
| NT vs NK | ARO:3004063 | EdeQ | peptide antibiotic; polyamine antibiotic | antibiotic inactivation | Edeine acetyltransferase | NK | 1.2644 | -20.2701 | 0.5285 | 0.0636 |
| NT vs NK | ARO:3004291 | Rhodobacter sphaeroides ampC beta-lactamase | cephalosporin; penam | antibiotic inactivation | ampC-type beta-lactamase | NK | 0.4882 | -1.9538 | 0.5285 | 0.1000 |
| NT vs NK | ARO:3004682 | aadA27 | aminoglycoside antibiotic | antibiotic inactivation | ANT(3'') | NK | 0.5584 | -19.0910 | 0.5285 | 0.0636 |
| NT vs NK | ARO:3006103 | OXA-917 | carbapenem; cephalosporin; penam | antibiotic inactivation | OXA beta-lactamase | NT | 1.1164 | 2.5779 | 0.5285 | 0.0765 |
| NT vs NK | ARO:3006688 | PDC-318 | monobactam; carbapenem; cephalosporin | antibiotic inactivation | PDC beta-lactamase | NK | 0.6426 | -19.2935 | 0.5285 | 0.0636 |
| NT vs NK | ARO:3006997 | SPS-1 | carbapenem | antibiotic inactivation | SPS beta-lactamase | NT | 0.6467 | 19.3028 | 0.5285 | 0.0636 |
| NT vs NK | ARO:3007050 | mreA | macrolide antibiotic | antibiotic efflux | major facilitator superfamily (MFS) antibiotic efflux pump | NT | 0.8014 | 1.5044 | 0.5285 | 0.1000 |
| ZT vs ZK | ARO:3000181 | tet(V) | tetracycline antibiotic | antibiotic efflux | major facilitator superfamily (MFS) antibiotic efflux pump | ZK | 0.4052 | -18.6282 | 0.4755 | 0.0636 |
| ZT vs ZK | ARO:3000215 | mecR1 | penam | antibiotic target replacement | methicillin resistant PBP2 | ZK | 1.8710 | -20.8354 | 0.4755 | 0.0636 |
| ZT vs ZK | ARO:3000319 | mphC | macrolide antibiotic | antibiotic inactivation | macrolide phosphotransferase (MPH) | ZK | 0.7422 | -3.3295 | 0.4755 | 0.0765 |
| ZT vs ZK | ARO:3000790 | mdsB | monobactam; carbapenem; cephalosporin; cephamycin; penam; phenicol antibiotic; penem | antibiotic efflux | resistance-nodulation-cell division (RND) antibiotic efflux pump | ZK | 2.5127 | -2.8398 | 0.4755 | 0.1000 |
| ZT vs ZK | ARO:3000801 | MexD | macrolide antibiotic; fluoroquinolone antibiotic; aminoglycoside antibiotic; cephalosporin; penam; tetracycline antibiotic; aminocoumarin antibiotic; diaminopyrimidine antibiotic; phenicol antibiotic | antibiotic efflux | resistance-nodulation-cell division (RND) antibiotic efflux pump | ZK | 1.6004 | -20.6100 | 0.4755 | 0.0636 |
| ZT vs ZK | ARO:3001302 | chrB | macrolide antibiotic; lincosamide antibiotic | antibiotic target alteration | non-erm 23S ribosomal RNA methyltransferase (G748) | ZK | 0.4454 | -2.9408 | 0.4755 | 0.0765 |
| ZT vs ZK | ARO:3001313 | facT | elfamycin antibiotic | antibiotic efflux | major facilitator superfamily (MFS) antibiotic efflux pump | ZK | 0.8169 | -1.5937 | 0.4755 | 0.1000 |
| ZT vs ZK | ARO:3001483 | OXA-205 | carbapenem; cephalosporin; penam | antibiotic inactivation | OXA beta-lactamase | ZK | 0.4528 | -1.8578 | 0.4755 | 0.0765 |
| ZT vs ZK | ARO:3001794 | OXA-45 | carbapenem; cephalosporin; penam | antibiotic inactivation | OXA beta-lactamase | ZK | 0.0542 | -15.7247 | 0.4755 | 0.0636 |
| ZT vs ZK | ARO:3001932 | CTX-M-71 | cephalosporin | antibiotic inactivation | CTX-M beta-lactamase | ZK | 0.3033 | -3.3174 | 0.4755 | 0.0765 |
| ZT vs ZK | ARO:3002510 | LRA-3 | cephalosporin; penam | antibiotic inactivation | subclass B3 LRA beta-lactamase | ZK | 0.4595 | -3.5011 | 0.4755 | 0.0765 |
| ZT vs ZK | ARO:3002817 | carA | macrolide antibiotic | antibiotic target protection | Miscellaneous ABC-F subfamily ATP-binding cassette ribosomal protection proteins | ZK | 3.9880 | -1.7883 | 0.4755 | 0.1000 |
| ZT vs ZK | ARO:3002970 | vanT gene in vanC cluster | glycopeptide antibiotic | antibiotic target alteration | glycopeptide resistance gene cluster; vanT | ZK | 1.2198 | -20.2182 | 0.4755 | 0.0636 |
| ZT vs ZK | ARO:3003016 | dfrA20 | diaminopyrimidine antibiotic | antibiotic target replacement | trimethoprim resistant dihydrofolate reductase dfr | ZK | 1.3286 | -20.3415 | 0.4755 | 0.0636 |
| ZT vs ZK | ARO:3003324 | Bacillus subtilis mprF | peptide antibiotic | antibiotic target alteration | defensin resistant mprF | ZK | 1.9670 | -4.3067 | 0.4755 | 0.1000 |
| ZT vs ZK | ARO:3003395 | Mycobacterium tuberculosis rpsL mutations conferring resistance to Streptomycin | aminoglycoside antibiotic | antibiotic target alteration | antibiotic-resistant rpsL | ZK | 2.8617 | -3.1053 | 0.4755 | 0.1000 |
| ZT vs ZK | ARO:3003681 | TriC | disinfecting agents and antiseptics | antibiotic efflux | resistance-nodulation-cell division (RND) antibiotic efflux pump | ZK | 0.7160 | -3.3034 | 0.4755 | 0.0765 |
| ZT vs ZK | ARO:3004143 | AxyX | macrolide antibiotic; fluoroquinolone antibiotic; aminoglycoside antibiotic; cephalosporin | antibiotic efflux | resistance-nodulation-cell division (RND) antibiotic efflux pump | ZT | 0.1743 | 3.2594 | 0.4755 | 0.0765 |
| ZT vs ZK | ARO:3005059 | LptD | carbapenem; peptide antibiotic; aminocoumarin antibiotic; rifamycin antibiotic | antibiotic efflux | ATP-binding cassette (ABC) antibiotic efflux pump | ZK | 0.5974 | -19.1884 | 0.4755 | 0.0636 |
